# Successful Therapy for a Patient With an Infected Ascending Aortic Graft and Sternal Osteomyelitis Without Graft Removal

**Published:** 2008-08-25

**Authors:** Dominik W. Schmid, Christina Orasch-Jörg, Reto Wettstein, Daniel F. Kalbermatten, Atanas Todorov, Gerhard Pierer

**Affiliations:** ^a^ Department of Plastic, Reconstructive and Aesthetic Surgery, University Hospital of Basel, Basel, Switzerland; ^b^ Division of Infectious Diseases and Hospital Epidemiology, University of Basel, Basel, Switzerland; ^c^ Department of Heart and Thoracic Surgery, University of Basel, Basel, Switzerland

## Abstract

**Objective:** Following open-heart surgery, sternal osteomyelitis or infection of the graft may be a serious complication with high mortality rates. The recommended treatment of an infected graft is its explantation. Because of the poor performance status of the patient, this may not always be an option. We report a successful treatment concept without removal of the infected graft. **Methods:** The infected ascending aortic graft and the remaining sternum of a critically ill 60-year-old man were covered with a bilateral pectoralis muscle flap. **Results:** Postoperatively, the laboratory test values normalized and the patient was discharged 1 month after the intervention. One year after surgery, the patient was in good condition and the examination showed no signs of infection. **Conclusion:** The thus demonstrated treatment concept with insertion of well-vascularized tissues in combination with a specific antibiotic regime in our hands proved to be an additional option for the successful management of life-threatening infections of a sternal osteomyelitis in combination of an infected aortic graft.

Following open-thoracic surgery, sternal osteomyelitis occurs in 3% to 5%^1^ and aortic graft infection in 0.9% to 6% of cases.^2^ The latter is a rare but serious complication with mortality rates ranging from 25% to 75%.^3^ Radical debridement and flap coverage is the treatment of choice for recalcitrant sternal osteomyelitis,^4^ whereas the recommended treatment of an infected graft is the explantation.^5^ On the basis of the poor condition of some of the patients, graft replacement is associated with high mortality rate (mean = 14%–80%)^6^ and thus coverage with muscle flaps has been suggested.^7^ We report the successful therapy of a case with both, an infected ascending aortic graft and sternal osteomyelitis, without removal of the graft.

## CASE REPORT

**Figure 1.**
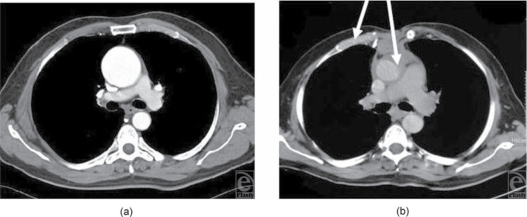
(a) Preoperative computed tomographic (CT) scan. (b) Postoperative (12 months) CT scan.

A 60-year-old man underwent ascending aorta replacement (Gelweave Valsalva 28, Vascutek Inc, Glasgow, United Kingdom) and aortic valve repair for a 62-mm aneurysm (Fig 1a). Because of acute postoperative bleeding, the aortic graft was replaced by a composite graft equipped with a mechanic aortic valve. On postoperative day 19, the patient developed a systemic inflammatory response syndrome with fever, leukocytosis, and tachycardia. The focus of infection appeared to be a sternal osteomyelitis with delayed healing of the sternotomy wound, purulent secretion, and perifocal erythema. Blood cultures and wound swabs were collected before empiric antibiotic therapy with cefepime was started. Growth of *Staphylococcus aureus* (sensitive to oxacillin) was found in all specimens. Sternal debridement was performed and the antibiotic therapy was changed to a combination therapy (flucloxacillin, rifampicin, and an aminoglycoside). A prosthetic valve endocarditis could not be excluded, although no typical vegetations were seen by the transesophageal echocardiography. After 2 weeks, a second sternal debridement was necessitated because of persistent wound secretion and dehiscence. The wound closure with a VAC (KCI International, the Netherlands) system was performed by the cardiovascular surgeon and an antibiotic therapy with only flucloxacillin and rifampicin was continued. Nevertheless, the patient developed fever and elevated white blood cells counts (Table 1). At this point, the removal and replacement of the probably infected composite graft was discussed and the plastic surgery team was consulted. The treatment strategy agreed upon consisted of a radical debridement and the introduction of well-vascularized tissue around the graft and the sternal wound (1) to fill dead space and (2) to increase the effect of antibiotics locally.

The preparation of the pectoralis muscle flaps as a turnover flap on the right side pedicled on the basis of perforators from the internal mammary artery, and an advancement flap pedicled on the thoracoacromial vessels on the left side, was followed by a subtotal sternal debridement. Both flaps were placed in the sternal wound and wrapped around the graft from both sides. The soft-tissue defect was closed primarily after placing 4 drains. Operation time was 280 minutes. Swabs taken intraoperatively from the graft showed growth of *Candida albicans* but absence of *S aureus*. Fluconazole was added to the antibiotic regime.

Following this intervention, infectious parameters decreased and hemodynamic and respiratory parameters had stabilized so that the patient could be extubated and could leave the intensive care unit 2 days later. One month after radical debridement and wrapping the bilateral muscle flaps around the graft, the patient was discharged.

Treatment with flucloxacillin 6 × 2 g IV per day and rifampicin 2 × 450 mg po per day for a total of 6 weeks, initially combined with amikacine 1g IV per day for 2 weeks, was stopped on the day of discharge.

The conclusion to replace the yeast-infected graft was reached interdisciplinary. For technical and clinical reasons, such as the size of the needed homograft and the poor condition of the patient, we decided to treat this patient conservatively with fluconazole 200 mg po per day (dose adapted to renal insufficiency).

The laboratory test values normalized (Table 1) and fluconazole was stopped after 8 months. Blood culture results 1 and 2 months after stopping the antibiotic therapy as well as 1 and 2 months after stopping fluconazole remained sterile. One year after surgery, the patient was in good condition. The examination showed no signs of infection as well as sufficient chest stability and good function of the upper extremity with slightly decreased strength of adduction. Respiratory functions remained unaltered compared with the preoperative status. The follow-up computed tomographic scan showed perfused muscle flaps around the ascending aortic graft and no evidence of mediastinal fluid collections (Fig 1b).

## DISCUSSION

Sternal osteomyelitis combined with an infection of a vascular graft is a serious complication with uncertain outcomes. The treatment of sternal osteomyelitis with muscle flaps is a well-established procedure but the combination with ascending aortic graft infection makes it more difficult. The best therapy would include the replacement of the graft. However, in a septic patient with respiratory and hemodynamic instabilities this is often impossible.

In the present case, we decided to cover the ascending aortic graft and the remaining sternum with well-vascularized tissue—a muscle flap—after a radical debridement. Potential donor muscle flaps are the rectus abdominis, the latissimus dorsi, or the serratus anterior flap. The greater omentum flap has also been recommended as a therapy option for a sternal osteomyelitis. However, in our opinion, one of the big advantages of covering the sternum with a muscle flap is the avoidance of laparotomy for harvesting of the greater omentum. Laparotomy poses an additional stress for the patient, especially if he is septic and critically ill.

Infections of proximal aortic grafts with *C albicans* are rare and usually associated with reduced immunocompetence, prolonged antibiotic therapy (like in our patient), extended parenteral nutrition, or IV drug abuse.^8^ Lifelong antifungal therapy has been recommended for this type of infection, but data supporting the optimal duration of antifungal therapy are lacking.^9^

With the procedure described in this article, we outline a treatment option for a combination of sternal osteomyelitis and infection of the ascending aortic graft without removal and replacement of the latter. One of the most important aspects in such complicated situations is an interdisciplinary cooperation of thoracic surgeons with infectious disease specialists and plastic surgeons, resulting in early diagnosis and optimal treatment—by antimicrobials as well as operative interventions—of the patient.

## Figures and Tables

**Table 1:** Pre- and postoperative laboratory test values

	**Preoperative**	**Postoperative, 3 wk**	**Postoperative, 4 mo**	**Postoperative, 1 y**
C-reactive protein	181 mg/L	21 mg/L	<8 mg/L	10 mg/L
Leukocytes	10.3 × 10^9^/L	7.5 × 10^9^/L	9.1 × 10^9^/L	8.1 × 10^9^/L
Albumin	14 g/L	24 g/L	42 g/L	41 g/L
